# Evaluation of Pulmonary Fibrosis Outcomes by Race and Ethnicity in US Adults

**DOI:** 10.1001/jamanetworkopen.2023.2427

**Published:** 2023-03-01

**Authors:** Ayodeji Adegunsoye, Elizabeth Freiheit, Emily N. White, Bhavika Kaul, Chad A. Newton, Justin M. Oldham, Cathryn T. Lee, Jonathan Chung, Nicole Garcia, Sahand Ghodrati, Rekha Vij, Renea Jablonski, Kevin R. Flaherty, Paul J. Wolters, Christine Kim Garcia, Mary E. Strek

**Affiliations:** Section of Pulmonary and Critical Care Medicine, Department of Medicine, The University of Chicago, Chicago, Illinois; Department of Biostatistics, School of Public Health, University of Michigan, Ann Arbor; Department of Biostatistics, School of Public Health, University of Michigan, Ann Arbor; Section of Pulmonary, Critical Care, Allergy and Sleep Medicine, Department of Medicine, University of California San Francisco; Division of Pulmonary and Critical Care Medicine, Department of Internal Medicine, University of Texas Southwestern, Dallas; Division of Pulmonary & Critical Care Medicine, Department of Medicine, University of Michigan, Ann Arbor; Section of Pulmonary and Critical Care Medicine, Department of Medicine, The University of Chicago, Chicago, Illinois; Department of Radiology, The University of Chicago, Chicago, Illinois; Section of Pulmonary and Critical Care Medicine, Department of Medicine, The University of Chicago, Chicago, Illinois; Division of Pulmonary, Critical Care, and Sleep Medicine, University of California, Davis, Sacramento; Section of Pulmonary and Critical Care Medicine, Department of Medicine, The University of Chicago, Chicago, Illinois; Section of Pulmonary and Critical Care Medicine, Department of Medicine, The University of Chicago, Chicago, Illinois; Division of Pulmonary & Critical Care Medicine, Department of Medicine, University of Michigan, Ann Arbor; Section of Pulmonary, Critical Care, Allergy and Sleep Medicine, Department of Medicine, University of California San Francisco; Division of Pulmonary, Allergy and Critical Care Medicine, Columbia University, New York, New York; Section of Pulmonary and Critical Care Medicine, Department of Medicine, The University of Chicago, Chicago, Illinois

## Abstract

**IMPORTANCE:**

Pulmonary fibrosis (PF) is characterized by progressive scarring of lung tissue and poor survival. Racial and ethnic minority populations face the greatest risk of morbidity and mortality from disparities impacting respiratory health, but the pattern of age at clinically relevant outcomes across diverse racial and ethnic populations with PF is unknown.

**OBJECTIVE:**

To compare the age at PF-related outcomes and the heterogeneity in survival patterns among Hispanic, non-Hispanic Black, and non-Hispanic White participants.

**DESIGN, SETTING, AND PARTICIPANTS:**

This cohort study included adult patients with a PF diagnosis and used data from prospective clinical registries: the Pulmonary Fibrosis Foundation Registry (PFFR) for the primary cohort and registries from 4 geographically distinct tertiary hospitals in the US for the external multicenter validation (EMV) cohort. Patients were followed between January 2003 and April 2021.

**EXPOSURES:**

Race and ethnicity comparisons between Black, Hispanic, and White participants with PF.

**MAIN OUTCOMES AND MEASURES:**

Age and sex distribution of participants were measured at the time of study enrollment. All-cause mortality and age at PF diagnosis, hospitalization, lung transplant, and death were assessed in participants over 14 389 person-years. Differences between racial and ethnic groups were compared using Wilcoxon rank sum tests, Bartlett 1-way analysis of variance, and χ^2^ tests, and crude mortality rates and rate ratios were assessed across racial and ethnic categories using Cox proportional hazards regression models.

**RESULTS:**

In total, 4792 participants with PF were assessed (mean [SD] age, 66.1 [11.2] years; 2779 [58.0%] male; 488 [10.2%] Black, 319 [6.7%] Hispanic, and 3985 [83.2%] White); 1904 were in the PFFR and 2888 in the EMV cohort. Black patients with PF were consistently younger than White patients (mean [SD] age at baseline, 57.9 [12.0] vs 68.6 [9.6] years; *P* < .001). Hispanic and White patients were predominantly male (Hispanic: PFFR, 73 of 124 [58.9%] and EMV, 109 of 195 [55.9%]; and White: PFFR, 1090 of 1675 [65.1%] and EMV, 1373 of 2310 [59.4%]), while Black patients were less likely to be male (PFFR, 32 of 105 [30.5%] and EMV, 102 of 383 [26.6%]). Compared with White patients, Black patients had a lower crude mortality rate ratio (0.57 [95% CI, 0.31–0.97), but for Hispanic patients, the mortality rate ratio was similar to that of White patients (0.89; 95% CI, 0.57–1.35). Mean (SD) hospitalization events per person were highest among Black patients compared with Hispanic and White patients (Black: 3.6 [5.0]; Hispanic, 1.8 [1.4]; and White, 1.7 [1.3]; *P* < .001). Black patients were consistently younger than Hispanic and White patients at first hospitalization (mean [SD] age: Black, 59.4 [11.7] years; Hispanic, 67.5 [9.8] years; and White, 70.0 [9.3] years; *P* < .001), lung transplant (Black, 58.6 [8.6] years; Hispanic, 60.5 [6.1] years; and White, 66.9 [6.7] years; *P* < .001), and death (Black, 68.7 [8.4] years; Hispanic, 72.9 [7.6] years; and White, 73.5 [8.7] years; *P* < .001). These findings remained consistent in the replication cohort and in sensitivity analyses within prespecified deciles of age groups.

**CONCLUSIONS AND RELEVANCE:**

In this cohort study of participants with PF, racial and ethnic disparities, especially among Black patients, were found in PF-related outcomes, including earlier onset of death. Further research is essential to identify and mitigate the underlying responsible factors.

## Introduction

Death rates from chronic respiratory diseases have recently increased, largely driven by the rising burden of interstitial lung diseases (ILDs) doubling mortality rates over the past 4 decades.^1,2^ Pulmonary fibrosis (PF), a form of ILD, is characterized by destruction of lung tissue and accounts for the highest increase in mortality rates.^3,4^ The disproportionate impact exerted by ILD on PF-related outcomes such as respiratory-related deaths is a function of its epidemiological burden, greater disease severity, and an increasingly aging population, culminating in widespread recognition of ILD as the foremost indication for lung transplant in the US.^5,6^

Racial and ethnic minority populations face the greatest risk of morbidity and mortality from health disparities and preexisting socioeconomic inequities.^7–9^ Black patients have high rates of respiratory impairment and more frequent pulmonary involvement with autoimmune disease, are 3 times as likely to die of obstructive lung diseases like asthma, and may have differential survival in ILD when compared with White individuals.^10–14^ While these disparities impact factors that span the spectrum from diagnosis to the time of death or lung transplant, poor enrollment of racial and ethnic minority individuals in ILD registries and clinical trials has limited our understanding of the interrelationship between health disparities and racial and ethnic differences in outcomes among patients with PF.

As PF is deemed more prevalent in White individuals,^15^ the age at which clinically relevant outcomes occur in racial and ethnic minority populations is less well understood. We hypothesized that the age at clinically relevant outcomes among patients with PF differs by racial or ethnic category. Therefore, our study sought to evaluate the age at PF-related outcomes (diagnosis, hospitalization, lung transplant, and death) and the heterogeneity in survival patterns among White, Black, and Hispanic participants with PF in a nationally acquired US registry and to validate these findings with data from 4 geographically disparate tertiary care centers with PF expertise.

## Methods

### Study Setting

This cohort study was conducted using the Pulmonary Fibrosis Foundation (PFF) patient registry as the primary cohort. Independent replication of findings was performed within an external multicenter validation (EMV) cohort of prospective registries from 4 geographically distinct tertiary hospitals. The PFF Registry (PFFR), acquired from the PFF Care Center Network (PFF-CCN), the largest nationwide consortium network of PF centers in the US, is a multicenter clinical registry of patients with ILD that contains data on more than 2000 patients across the US collected since 2016.^16^ All registry resources used from the primary and replication cohorts contain details on ILD diagnosis, lung function indices, radiographic data, and clinical course for enrolled patients. All patients provided written informed consent at the time of center registry enrollment. This study was approved by the respective institutional review board for each participating center and followed the Strengthening the Reporting of Observational Studies in Epidemiology (STROBE) reporting guideline.^17^

### Study Population and Design

We performed a retrospective analysis of prospectively enrolled consenting patients with a multidisciplinary PF diagnosis including idiopathic PF (IPF), connective tissue disease–related ILD (CTD-ILD), fibrotic hypersensitivity pneumonitis (fHP), and unclassifiable or other subtypes such as pulmonary sarcoidosis, pneumoconiosis, and pleuroparenchymal fibroelastosis within the PFFR (March 2016 through February 2020), which was the primary cohort, and 4 tertiary hospital registries, which composed the EMV replication cohort: The University of Chicago, The University of Texas Southwestern Medical Center, University of California San Francisco, and University of California, Davis (January 2003 through April 2021). Given the heterogeneity of PF, our study was designed to assess findings in a replication cohort using currently available clinical data from geographically disparate sources without the constraints of more restrictive criteria within the national PFFR, where patient enrollment was enriched for specific PF subtypes such as IPF. The design of the PFF-CCN and PFFR has been previously published.^16,18^ Patients in the EMV replication cohort were enrolled in their hospital registries at ILD diagnosis; therefore, all those concurrently enrolled in the PFFR were excluded from the EMV replication cohort to avoid duplications. Data on PF-specific therapy for the University of California, Davis, cohort was unavailable at the time of institutional review board study approval. To compare clinically relevant outcomes among patients with PF, categorization of self-reported race and ethnicity was implemented per federally defined US Census Bureau standards on race (American Indian or Alaska Native, Asian, Black or African American, Native Hawaiian or Pacific Islander, and White) and ethnicity (Hispanic or not Hispanic).^19^ Patients in 1 of 3 predefined racial and ethnic categories—Black (not Hispanic), Hispanic, or White (not Hispanic)—were included in the analysis.

### Follow-up and Study Outcomes

Patients entered the study cohorts on the date of registry enrollment, and all patients were followed up until occurrence of death, lung transplant, the end of the study period, or loss to follow-up. Patients were censored if alive without transplant at the end of the study period or when lost to follow-up. Person-time was averaged at 30 days per month from registry enrollment to study end point. We quantified clinically relevant milestones of interest in the natural history of PF, including ILD diagnosis, hospitalizations, lung transplant, and all-cause mortality. We compared age at occurrence of these outcomes across racial and ethnic categories.

### Statistical Analysis

We summarized baseline characteristics using descriptive statistics and present these as means with SDs, medians with IQRs, or counts with proportions, as appropriate. The primary aim was to determine if age at PF-related outcomes and survival patterns differed across Black, Hispanic, and White patients with PF. We compared differences in clinical milestones, time to event, and outcomes between racial and ethnic groups using Wilcoxon rank sum tests, the Bartlett 1-way analysis of variance, and χ^2^ tests, as indicated. We assessed crude mortality rates and rate ratios across racial and ethnic categories. In assessment of age at PF-related outcomes substratified by age group in deciles (<50, 50–59, 60–69, and 70–80 years), the primary and replication cohorts were analyzed separately; then a meta-analysis across both cohorts was performed.

To assess whether racial and ethnic categories were associated with differential transplant-free survival from time of enrollment, Poisson generalized linear models with a logistic regression link were first used to assess mortality incidence rate ratios across Black, Hispanic, and White patients; then we constructed multivariable Cox proportional hazards regression models with robust variances for hazard ratio (HR) estimation. Survival curves were plotted using the Kaplan-Meier survival estimator, and the log-rank test was used for group comparisons. The HRs for death are reported with study participants of White race and ethnicity as the reference group. In all Cox proportional hazards regression analyses, mortality and lung transplant were considered a composite outcome. All multivariable Cox models were adjusted for potential confounders selected a priori. These were known prognostic determinants in PF, such as age, sex, PF subtype, and physiologic indices of disease severity such as forced vital capacity (FVC), diffusing capacity of the lung for carbon monoxide (DLCO), and Gender-Age-Physiology Index (GAP) score (possible range, −2 to 8, with higher scores indicating worse prognosis). Outcome modeling incorporated a random effect to account for heterogeneity across hospitals as well as clustering of racial and ethnic groups within hospitals. Given the study hypothesis that differences across racial and ethnic groups would exist in 4 main temporal domains (time to diagnosis, time to hospitalization, time to lung transplant, and time to death), 2-sided, Bonferroni-corrected *P* < .05 was considered statistically significant.

In sensitivity analyses, we performed patient stratification by center to assess consistency of study results across sites. Quintile stratification of age groups was performed in deciles and categorized by race and ethnicity, and the Jonckheere-Terpstra nonparametric test was used to assess the trend in age across ordered racial and ethnic groups within the pooled population. Analyses were performed without imputation, as missing covariates were infrequent in our cohort (<5%). Postestimation tests demonstrated goodness of fit for all models. We tested the proportional hazards assumption by examining covariates over time and by regressing Schoenfeld residuals over time in the Cox proportional hazards regression survival models, and all models evaluated passed this test. All analyses were conducted using Stata, version 17 (StataCorp LLC), and code is available upon request.

## Results

### Patient Characteristics

We identified 5275 patients with a diagnosis of PF from January 2003 through April 2021 within the study population, of whom 4792 patients (90.8%), assessed over 14 389 person-years, met eligibility criteria (mean [SD] age, 66.1 [11.2] years; 488 [10.2%] Black, 319 [6.7%] Hispanic, and 3985 [83.2%] White), including 1904 in the primary cohort and 2888 in the replication cohort (Table 1 and eFigure 1 and eTable 1 in Supplement 1). The mean (SD) age at enrollment of patients into the PFFR was 67.8 (10.1) years, while the mean (SD) age for the EMV replication cohort was 65.0 (11.9) years. Most enrolled study participants were men (overall: 2779 [58.0%] male, 2013 [42.0%] female; PFFR: 1195 [62.8%] male, 709 [37.2%] female; EMV: 1584 [54.8%] male, 1304 [45.2%] female), had previously smoked tobacco, and were overweight. Lung function was moderately impaired in the PFFR cohort, with a mean (SD) percent predicted FVC of 68.5 (18.1), percent predicted DLCO of 42.7 (17.3), and GAP score of 3.4 (2.0). These data were similar to those of the EMV cohort except for a slightly higher mean (SD) percent predicted DLCO of 51.4 (21.6). In both cohorts, the most frequent PF subcategory was IPF, and a substantial minority had received antifibrotic therapy.

### Differences Across Racial and Ethnic Groups at Study Enrollment

Black patients diagnosed with PF were consistently younger than Hispanic and White patients (mean [SD] age at baseline in the PFFR: Black, 57.9 [12.0] years; Hispanic, 65.4 [10.6] years; White, 68.6 [9.6] years; *P* < .001; EMV: Black, 57.3 [13.6] years; Hispanic, 61.4 [12.3] years; White, 66.6 [11.0] years; *P* < .001). Black patients with PF were least likely to be male (PFFR, 32 of 105 [30.5%]; EMV, 102 of 383 [26.6%]) and had the highest mean (SD) body mass index (calculated as weight in kilograms divided by height in meters squared) (PFFR, 31.0 [7.8]; EMV, 30.1 [7.1]). In contrast, Hispanic and White patients were predominantly male (PFFR: Hispanic, 73 of 124 [58.9%]; White, 1090 of 1675 [65.1%]; EMV: Hispanic, 109 of 195 [55.9%]; White, 1373 of 2310 [59.4%]), and White patients had the highest prevalence of tobacco smokers (PFFR, 1008 of 1675 [60.2%]; EMV, 1370 of 2278 [60.1%]). When assessing lung function impairment across racial and ethnic groups, Black patients had the lowest mean (SD) percent predicted DLCO (PFFR: 40.0 [23.0]; EMV, 48.9 [22.1]) and GAP scores (PFFR, 1.9 [2.1]; EMV, 3.0 [1.5]), while the mean (SD) percent predicted FVC was highest in White patients (PFFR, 69.0 [17.8]; EMV, 69.0 [19.0]) and lower in Hispanic patients (PFFR, 60.9 [17.0]; EMV, 64.8 [18.7]).

The PF subcategories and specific therapies varied by racial category in both cohorts. For example, IPF was most prevalent in White patients, whereas fHP was most prevalent in Hispanic patients. Black patients had the highest prevalence of CTD-ILD in the PFFR (Black, 57 of 105 [54.3%]; Hispanic, 27 of 124 [21.8%]; White, 227 of 1675 [13.6%]; *P* < .001) and the EMV cohort (Black, 148 of 383 [38.6%]; Hispanic, 53 of 195 [27.2%]; and White, 290 of 2310 [12.6%]; *P* < .001). Further, Black patients had the highest prevalence of unclassifiable or other PF subcategories. The proportion of patients treated with antifibrotic therapy was higher among Hispanic and White patients, while Black patients had the highest proportion of corticosteroid therapy.

### Association of Race and Ethnicity With PF Outcomes

The median follow-up time for the PFFR cohort was 2.4 years (IQR, 1.5–3.1 years), during which 397 deaths and 185 lung transplants occurred, while the EMV cohort had a longer median follow-up time at 3.4 years (IQR, 1.4–7.5 years), during which 1042 deaths and 250 lung transplants occurred. A minority of patients in both cohorts were censored at loss to follow-up (PFFR: 223 of 1904 [11.7%] [Black, 8 of 105 (7.6%); Hispanic, 16 of 124 (12.9%); White, 199 of 1675 (11.9%)]; EMV: 452 of 2888 [15.7%] [Black, 55 of 383 (14.4%); Hispanic, 61 of 195 (31.3%); and White, 336 of 2310 (14.5%)]). Black patients had the longest mean survival time and the lowest number of deaths per 100 person-years. When compared with White patients, Black patients consistently had a lower crude mortality rate ratio (PFFR: 0.57; 95% CI, 0.31–0.97; *P* = .03; EMV: 0.56; 95% CI, 0.46–0.68; *P* < .001). Conversely, the mortality rate ratio among Hispanic patients (PFFR: 0.89; 95% CI, 0.57–1.35; *P* = .61; EMV: 0.91; 95% CI, 0.74–1.11; *P* = .36) was similar to that of White patients (Table 2). Similarly, the unadjusted HR for death was lower in Black patients, but similar in Hispanic patients, when compared with White patients (eTable 2 in Supplement 1). In adjusted Cox proportional hazards regression models, the mortality for all 3 racial and ethnic categories was similar within the PFFR; however, in the EMV cohort, the adjusted mortality HR remained lower in Black patients when compared with White patients.

### Temporal Patterns and Rates of Outcomes Across Racial and Ethnic Groups

**Figure 1.**
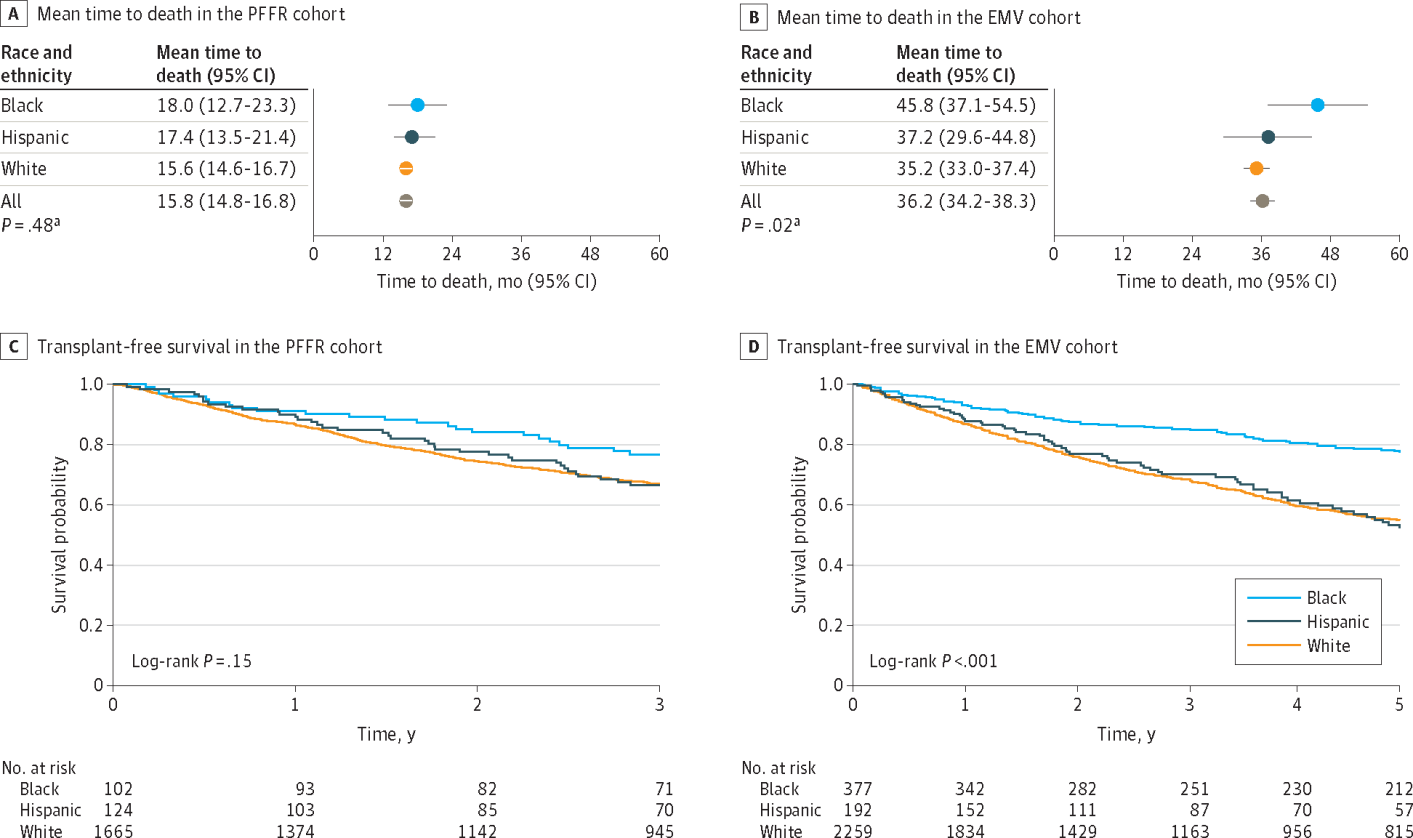
Survival Patterns Stratified by Racial and Ethnic Category PFFR indicates Pulmonary Fibrosis Foundation Registry; EMV, external multicenter validation. ^a^ *P* values are for the Bartlett 1-way analysis of variance test for equal variances comparing all 3 racial groups.

Among decedents in the PFFR, the mean (SD) time to death did not differ significantly among Black (18.0 [9.2] months), Hispanic (17.4 [9.2] months), and White participants (15.6 [10.0] months) (*P* = .48). This was in contrast to the EMV cohort, where the mean (SD) time to death was longest in Black decedents (45.8 [41.2] months; *P* = .02) but similar between Hispanic and White decedents (37.2 [30.5] months and 35.2 [32.9] months, respectively) (Figure 1A and B). Kaplan-Meier analyses suggested improved survival patterns in Black patients in both the EMV and PFFR cohorts (Figure 1C and D). The proportion of patients who received a lung transplant was lower in Black patients than Hispanic or White patients (PFFR: Black, 9 of 105 [8.6%]; Hispanic, 14 of 124 [11.3%]; White, 162 of 1675 [9.7%]; *P* = .77; EMV: Black, 21 of 374 [5.6%]; Hispanic, 18 of 164 [11.0%]; and White, 219 of 2117 [10.3%]; *P* = .02). Although hospitalizations were frequent across patients from all racial and ethnic categories, during the study period Black patients had the highest mean (SD) number of hospitalization events per person, while mean (SD) hospitalizations per person were less frequent in Hispanic and White patients (PFFR: Black, 3.6 [5.0]; Hispanic, 1.8 [1.4]; White, 1.7 [1.3]; *P* < .001; EMV: Black, 2.6 [2.2]; Hispanic, 1.9 [1.7]; White, 1.8 [1.4]; *P* < .001).

### Differences in Age at Outcomes Across Racial and Ethnic Groups With PF

**Figure 2.**
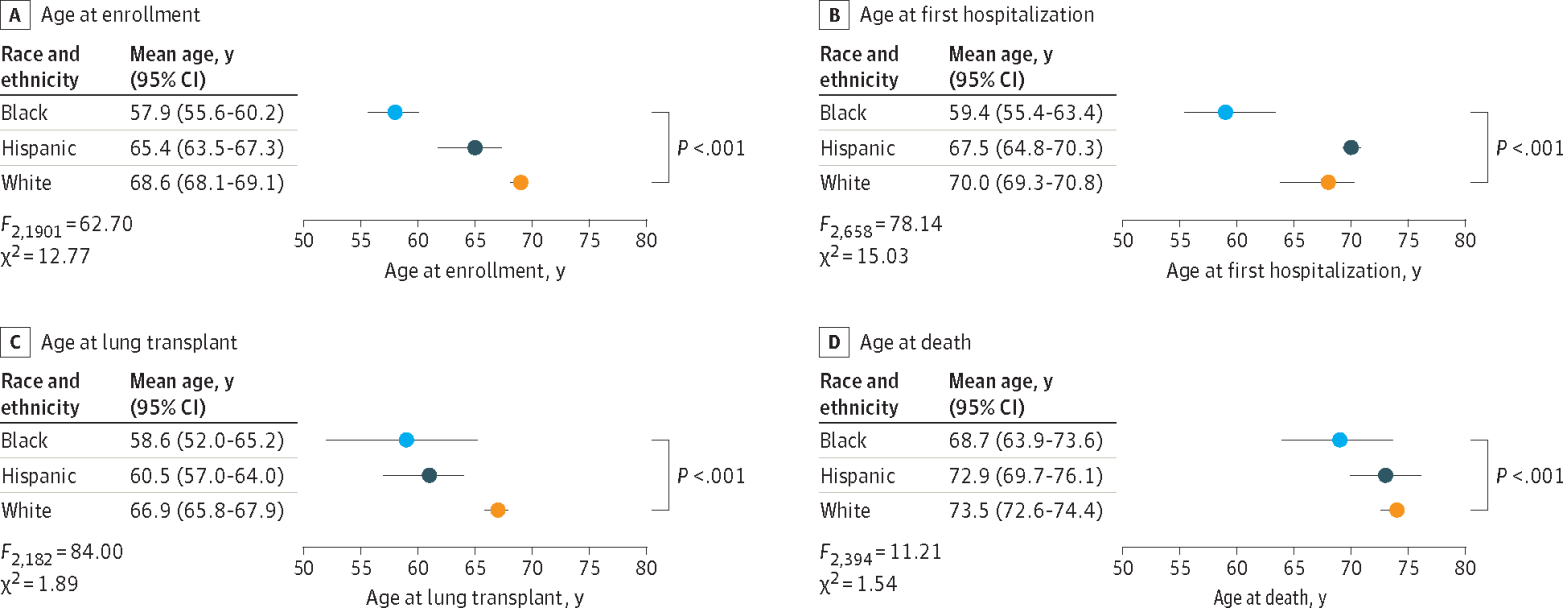
Mean Age at Outcomes Among Study Participants With Pulmonary Fibrosis, Stratified by Race and Ethnicity *P* values are for analysis of variance comparing all 3 racial groups.

Paralleling the disparities in age at study enrollment, Black patients had the youngest mean (SD) age at first hospitalization with PF when compared with Hispanic and White patients in the PFFR (Black, 59.4 [11.7] years; Hispanic, 67.5 [9.8] years; White, 70.0 [9.3] years; *P* < .001) and the EMV cohort (Black, 59.7 [12.3] years; Hispanic, 64.9 [8.2] years; White, 64.6 [9.3] years; *P* = .008) (Figure 2A and B and eFigure 2 in Supplement 1). These age differences between Black patients compared with Hispanic or White patients were mostly preserved through the clinical course of disease such that at the time of lung transplant, Black patients who received lung transplants remained youngest in contrast to Hispanic and White patients in the PFFR (mean [SD] age: Black, 58.6 [8.6] years; Hispanic, 60.5 [6.1] years; and White, 66.9 [6.7] years; *P* < .001) and the EMV cohort (Black, 52.0 [8.5] years; Hispanic, 61.8 [9.6] years; and White, 62.8 [7.1] years; *P* < .001) (Figure 2C and eFigure 2 in Supplement 1). With disease progression, these age disparities persisted until the time of death such that among the subpopulation of decedents, Black patients were consistently younger compared with Hispanic and White patients in the PFFR (mean [SD] age: Black, 68.7 [8.4] years; Hispanic, 72.9 [7.6] years; and White, 73.5 [8.7] years; *P* < .001) and the EMV cohort (Black, 65.0 [13.1] years; Hispanic, 68.2 [10.9] years; and White, 71.9 [9.2] years; *P* < .001) (Figure 2D and eFigure 2 in Supplement 1).

**Figure 3.**
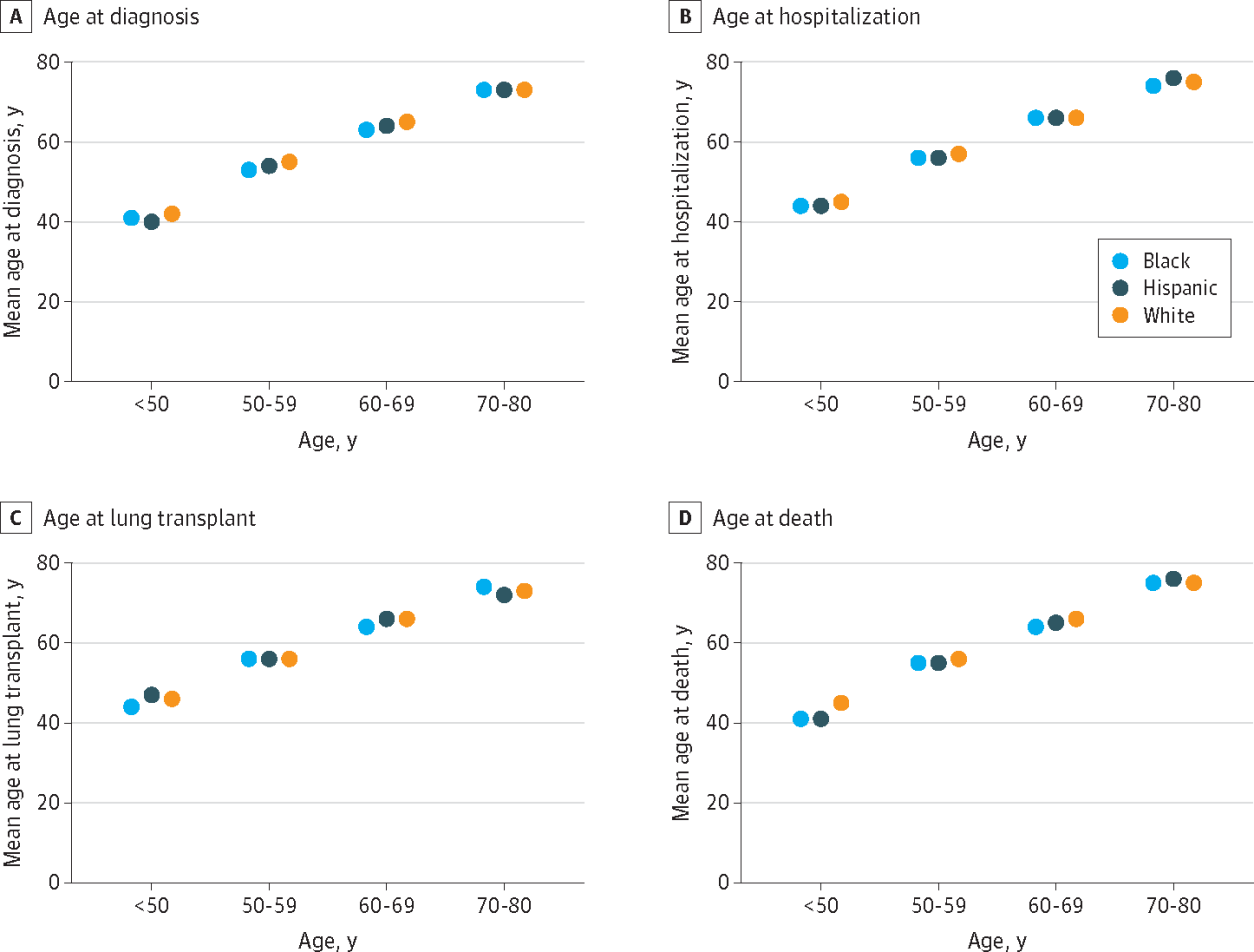
Distribution of Age at Outcomes Among Participants With Pulmonary Fibrosis, Stratified by Race and Ethnicity

In sensitivity analyses, assessment for effect modification by quintile stratification of age groups within racial and ethnic categories showed consistency with aggregated results for all PF outcomes analyzed (Figure 3 and eFigure 3 in Supplement 1). Black patients were consistently younger than White patients within the majority of age group deciles, while Hispanic patients were intermediate in age rankings. These findings remained consistent when the median age at PF outcomes across Black, Hispanic, and White patients was analyzed. Given the greater prevalence of CTD-ILD and lower prevalence of IPF among Black patients in both cohorts, we performed additional sensitivity analyses in which we stratified by IPF and non-IPF and by CTD-ILD and non–CTD-ILD subtypes of PF. In both cohorts, the age at outcomes consistently trended with the aggregated results for all PF outcomes analyzed (eFigures 4 through 8 in Supplement 1).

## Discussion

This study demonstrated substantial racial and ethnic disparities in PF outcomes over the life span of affected racial and ethnic minority individuals. Most notably, Black patients with PF were diagnosed and hospitalized, underwent lung transplant, and died at a younger age than Hispanic and White patients. Mortality rates also appeared to be lower in Black individuals than Hispanic and White individuals. However, any modest gain in life expectancy presumably accrued by Black patients fell short of the age differences, considering that PF diagnosis occurred a decade earlier in Black compared with White patients. The onset of debility attributable to PF generally occurs around middle age, and the median age at PF diagnosis typically occurs between the ages of 60 and 70 years.^20^ Thus, earlier onset of disease likely exerts considerable consequences on quality of life, hospitalization frequency, and functional capacity of affected persons. Black individuals reportedly often experience a delay in time to disease diagnosis,^21,22^ raising concern that even the apparent earlier PF diagnosis in Black patients might be delayed. Consistent with current trends in national epidemiological data in which racial and ethnic minority populations have lower waiting list access to lung transplants^23^ and are more frequently hospitalized,^24^ our study showed lower lung transplant rates and disproportionately higher hospitalization rates among Black and Hispanic patients with PF compared with White patients, further underscoring the impact of health care disparities on these racial and ethnic minority populations.

Our findings also reveal intriguing demographic patterns across racial and ethnic groups. In lockstep with the male predominance of the overall study population, most White patients with PF were men, contrasting with the female predominance in the Black subpopulation. In tandem with this divergent sex distribution, PF diagnostic subcategories differed in their patterns of distribution across racial and ethnic groups. The predominant diagnosis in White patients was IPF, while fHP was most commonly diagnosed among Hispanic patients and CTD-ILD was 3 to 4 times more common in Black patients compared with White patients. As autoimmune disease is more frequently reported in Black individuals^25,26^ and pet birds are often present in Hispanic households,^27^ this raises the possibility that confirmation bias in the medical diagnosis–making process results in the observed diagnostic differences across racial and ethnic groups.^28–31^ For example, in a study that examined US decedents with IPF within the National Center for Health Statistics database, Black decedents were less likely to be coded with IPF than were White decedents.^15^ Conversely, Hispanic decedents were more likely to be coded with IPF at the time of death.^15^ This idiosyncrasy necessitates the use of clinical data from geographically disparate centers with ILD expertise and carefully curated data sets that prioritize diagnostic ascertainment for assessing outcome disparities across racial and ethnic subgroups.

Black race, in particular, has been associated with increased symptomatic burden, frequent hospitalizations, and reduced life expectancy in numerous respiratory diseases.^10–13^ Unlike Hispanic and White patients, Black patients with PF in the current study had a notable increase in their survival time after diagnosis. These findings were similar to those previously described in a multicenter cohort of patients with various forms of ILD, in which race was demonstrated as an independent factor associated with survival,^14^ raising the possibility of lead-time bias, as earlier recognition of symptomatic disease could conceivably prompt the institution of medically necessary interventions. However, this potential benefit may have been constrained by previously well-described socioeconomic factors that disproportionately impact racial and ethnic minority populations,^32,33^ such that any improvement in survival time is insufficient to offset the earlier age at death. Unsurprisingly, the intermediate survival time observed in Hispanic patients may have resulted from their ethnic categorization as distinct from race, since these patients may have been Black Hispanic or White Hispanic patients.

The disparities in age at diagnosis of PF raise several questions that demand answers: What underlying pathophysiologic processes drive the earlier onset of PF in Black patients? Is the greater prevalence of autoimmunity in Black individuals an epiphenomenon, or does it truly drive PF onset? Why is it that earlier disease recognition and intervention do not completely abrogate the age disparities and lead to similar age at terminal end points? Ultimately, there is unquestionably a need for greater enrollment of racial and ethnic minority populations into registries to help us begin to find meaningful answers to these questions. Concerted efforts toward this might comprise broadening enrollment sites to include institutions that provide care to underserved and racial and ethnic minority populations, improving access to high-quality primary and subspecialty pulmonology care, and mitigating factors that perpetuate these racial differences in PF-related outcomes.^33^

### Limitations

Our study has several limitations. First, the PF subcategory of CTD-ILD within the Black cohort was 3 to 4 times larger than that of the White cohort, and idiopathic PF was comparatively less frequent among Black patients compared with White patients. The disproportionately greater incidence of CTD-ILD among Black patients may have influenced the prevalence of hospitalizations and lung transplant. Although our statistical survival models were adjusted for diagnostic subtypes of PF, it is possible that differences in the underlying PF subcategories likely led to the younger age at presentation, at hospitalization, and at death among Black patients. Similarly, varying densities of specific ethnic and racial groups within registries and the greater female prevalence among Black patients may have influenced the crude mean age at outcomes. However, the retrospective nature of this investigation limits our ability to infer causality in any of the relationships assessed. Further, even after substratifying the cohorts by PF subtype, the trend of lower age at outcomes among Black compared with White patients remained consistent. Second, as we were unable to conduct ascertainment of genetic ancestry, we used patients’ self-identified race and ethnicity as documented within the registries. Because race is a complex social construct that frequently reflects an individual’s perception of their familial origin and sociocultural environment, “race and ethnicity” as defined in the context of this study may be a surrogate for several unmeasured confounders. Third, conceivably, there may have been differences between dates of diagnosis and enrollment at a few PFF-CCN centers. However, because PF diagnosis and registry enrollment typically occur contemporaneously for most PFFR sites and diagnosis reascertainment was systematically performed at enrollment, the time of enrollment was uniformly accepted as the time of diagnosis across all study cohorts. Fourth, available follow-up time for the PFFR was constrained to 3 years, potentially limiting our ability to fully assess long-term survival. However, the estimates of our mortality outcomes were highly consistent across the primary and replication cohorts, reinforcing confidence in our results.

## Conclusions

The findings of this cohort study suggest that racial disparities in PF may be associated with earlier onset of terminal outcomes among racial and ethnic minority populations, especially Black patients. The disparities in age were pervasive and ran through the natural history of PF from diagnosis through disease progression, culminating in early occurrence of hospitalization, lung transplant, and death among racial and ethnic minority populations. Further research is essential to identify and mitigate the underlying responsible factors.

## Supplementary Material

Supplement 1

Supplement 2

## Figures and Tables

**Table 1.** Baseline Characteristics of the Study Population, Stratified by Race and Ethnicity

Characteristic	Patients^a^				*P* value
	Black (n = 488)	Hispanic (n = 319)	White (n = 3985)	All (N = 4792)	
**PFFR cohort**					
Patients, No.	105	124	1675	1904	
Age, mean (SD), y	57.9 (12.0)	65.4 (10.6)	68.6 (9.6)	67.8 (10.1)	<.001
Sex					
Female	73 (69.5)	51 (41.1)	585 (34.9)	709 (37.2)	<.001
Male	32 (30.5)	73 (58.9)	1090 (65.1)	1195 (62.8)	
Ever smoker	47 (44.8)	70 (56.5)	1008 (60.2)	1125 (59.1)	.006
BMI, mean (SD)	31.0 (7.8)	29.9 (5.5)	29.4 (5.7)	29.5 (5.9)	.02
Lung function measure, mean (SD)					
FVC, % predicted	68.2 (21.7)	60.9 (17.0)	69.0 (17.8)	68.5 (18.1)	<.001
DLCO, % predicted	40.0 (23.0)	42.5 (14.9)	42.9 (17.1)	42.7 (17.3)	.25
GAP score^b^	1.9 (2.1)	2.8 (1.9)	3.6 (1.9)	3.4 (2.0)	<.001
PF subcategory					
IPF	17 (16.2)	60 (48.4)	1102 (65.8)	1179 (61.9)	<.001
CTD-ILD	57 (54.3)	27 (21.8)	227 (13.6)	311 (16.3)	<.001
fHP	6 (5.7)	15 (12.1)	126 (7.5)	147 (7.7)	.16
Unclassifiable or other	25 (23.8)	22 (17.7)	220 (13.1)	267 (14.0)	.004
PF-specific therapy					
Antifibrotic	9 (8.6)	33 (26.6)	646 (38.6)	688 (36.1)	<.001
Corticosteroids	17 (16.2)	6 (4.8)	110 (6.6)	133 (7.0)	.001
None	462 (94.7)	280 (87.8)	3229 (81.0)	3971 (82.9)	<.001
Supplemental oxygen	42 (40.0)	67 (54.0)	741 (44.2)	850 (44.6)	.07
**EMV cohort**					
Patients, No.	383	195	2310	2888	
Age, mean (SD), y	57.3 (13.6)	61.4 (12.3)	66.6 (11.0)	65.0 (11.9)	<.001
Sex					
Female	281 (73.4)	86 (44.1)	937 (40.6)	1304 (45.2)	<.001
Male	102 (26.6)	109 (55.9)	1373 (59.4)	1584 (54.8)	
Ever smoker	168 (44.6)	82 (42.3)	1370 (60.1)^c^	1620 (56.9)	<.001
BMI, mean (SD)	30.1 (7.1)	27.9 (6.1)	29.1 (6.0)	29.1 (6.1)	.005
Lung function measure, mean (SD)					
FVC, % predicted	59.5 (17.2)	64.8 (18.7)	69.0 (19.0)	67.5 (19.1)	<.001
DLCO, % predicted	48.9 (22.1)	49.2 (22.2)	51.9 (21.5)	51.4 (21.6)	.03
GAP score^b^	3.0 (1.5)	3.0 (1.6)	3.1 (1.6)	3.1 (1.6)	.17
PF subcategory					
IPF	29 (7.8)	80 (41.5)	1034 (45.5)	1143 (39.6)	<.001
CTD-ILD	148 (38.6)	53 (27.2)	290 (12.6)	491 (17.0)	<.001
fHP	23 (6.0)	28 (14.4)	321 (13.9)	372 (12.9)	<.001
Unclassifiable or other	173 (45.2)	32 (16.4)	630 (27.3)	835 (28.9)	<.001
PF-specific therapy^d^					
Antifibrotic	14 (3.9)	32 (20.7)	411 (20.9)	457 (18.4)	<.001
Corticosteroids	267 (74.6)	70 (45.2)	916 (46.8)	1253 (50.7)	<.001
None	102 (26.7)	93 (47.7)	983 (42.6)	1178 (40.8)	<.001

Abbreviations: BMI, body mass index (calculated as weight in kilograms divided by height in meters squared); CTD-ILD, connective tissue disease– associated interstitial lung disease; DLCO, diffusing capacity of the lungs for carbon monoxide; EMV, external multicenter validation; fHP, fibrotic hypersensitivity pneumonitis; FVC, forced vital capacity; GAP, Gender-Age-Physiology Index; ILD, interstitial lung disease; IPF, idiopathic pulmonary fibrosis; PF, pulmonary fibrosis; PFFR, Pulmonary Fibrosis Foundation Registry.

a Categorical variables are presented as the number (percentage) of patients; continuous variables are presented as the mean (SD).

b Score range, −2 to 8, with higher scores indicating worse prognosis.

c Exception for participants in the EMV cohort: ever smoker, n = 2849.

d Data on the PF-specific therapy were unavailable for the University of California, Davis, subgroup of the EMV cohort.

**Table 2.** Association of Racial and Ethnic Categories With Pulmonary Fibrosis Outcomes in the PFFR and EMV Cohorts

Outcome	Patients			*P* value
	Black	Hispanic	White	
**PFFR cohort**				
Patients, No.	105	124	1675	NA
Survival time, mean (95% CI), mo^a^	33 (32–35)	32 (30–34)	31 (31–32)	.09
Deaths, No. (%)	14 (13.3)	24 (19.4)	359 (21.4)	.13
Crude mortality rate, events/100 person-years	5.4	8.4	9.4	<.001
Mortality rate ratio (95% CI)	0.57 (0.31–0.97)	NA	1 [Reference]	.03
	NA	0.89 (0.57–1.35)	1 [Reference]	.61
Unadjusted HR (95% CI)	0.65 (0.43–0.98)	NA	1 [Reference]	.04
	NA	0.98 (0.70–1.36)	1 [Reference]	.88
Adjusted HR (95% CI)^b^	1.03 (0.67–1.57)	NA	1 [Reference]	.91
	NA	1.12 (0.80–1.56)	1 [Reference]	.52
Participants hospitalized, No. (%)	36 (34.3)	50 (40.3)	575 (34.3)	.40
Hospitalizations per person, mean (SD), No.	3.6 (5.0)	1.8 (1.4)	1.7 (1.3)	<.001
Lung transplant, No. (%)	9 (8.6)	14 (11.3)	162 (9.7)	.77
**EMV cohort**				
Patients, No.	383	195	2310	NA
Survival time, mean (95% CI), mo^a^	182 (169–195)	98 (84–112)	115 (110–121)	<.001
Deaths, No. (%)^c^	89/374 (23.8)	64/164 (39.0)	889/2117 (42.0)	<.001
Crude mortality rate, events/100 person-years	3.5	8.5	8.6	<.001
Mortality rate ratio (95% CI)	0.56 (0.46–0.68)	NA	1 [Reference]	<.001
	NA	0.91 (0.74–1.11)	1 [Reference]	.36
Unadjusted HR (95% CI)	0.45 (0.37–0.55)	NA	1 [Reference]	<.001
	NA	1.01 (0.81–1.27)	1 [Reference]	.91
Adjusted HR (95% CI)^b^	0.68 (0.55–0.84)	NA	1 [Reference]	<.001
	NA	0.82 (0.65–1.03)	1 [Reference]	.09
Participants hospitalized, No. (%)^d^	93/183 (50.8)	27/70 (38.6)	195/807 (24.2)	<.001
Hospitalizations per person, mean (SD)	2.6 (2.2)	1.9 (1.7)	1.8 (1.4)	<.001
Lung transplant, No. (%)^c^	21/374 (5.6)	18/164 (11.0)	219/2117 (10.3)	.02

Abbreviations: EMV, external multicenter validation; HR, hazard ratio; NA, not applicable; PFFR, Pulmonary Fibrosis Foundation Registry.

a Computed from the point of registry enrollment.

b Cox proportional hazards regression models adjusted for age, sex, forced vital capacity, diffusing capacity of the lungs for carbon monoxide, interstitial lung disease subtype, and hospital center.

c Exception for data availability on lung transplantation and decedents for the EMV cohort; n = 2655.

d Exception for data availability on hospitalization for EMV cohort; n = 1094.

## Key Points

### Question

Does the age at onset of pulmonary fibrosis–related outcomes differ among Black, Hispanic, and White adults?

### Findings

In this cohort study of 4792 participants with pulmonary fibrosis across the US between January 2003 and April 2021, Black patients were significantly younger than Hispanic and White patients at the time of first hospitalization, lung transplant, and death. The number of hospitalizations was also higher among Black patients vs Hispanic and White patients.

### Meaning

These findings suggest that racial and ethnic minority populations may experience disparities in pulmonary fibrosis–related outcomes; policy initiatives and interventions for pulmonary fibrosis should consider factors underlying these disparities.
