# Supplementary material for: Evaluation of Pulmonary Fibrosis Outcomes by Race and Ethnicity in US Adults
Source: JAMA Netw Open. Author manuscript; Available in PMC 2024 Apr 1. (PMC10984340; doi:10.1001/jamanetworkopen.2023.2427)
Supplement: Supplement 1 [file NIHMS1973253-supplement-Supplement_1.pdf]

Supplementary Online Content

Adegunsoye A, Freiheit E, White EN, et al. Evaluation of pulmonary fibrosis outcomes by race and ethnicity in US adults. *JAMA Netw Open*. 2023;6(3):e232427. doi:10.1001/jamanetworkopen.2023.2427

eTable 1. Baseline Characteristics of the External Multicenter Validation (EMV) Cohort Stratified by Site

eTable 2. Association of Racial/Ethnic Categories With Mortality in Pulmonary Fibrosis in the External Multicenter Validation (EMV) Cohort

eFigure 1. STROBE Diagram

eFigure 2. Mean Age at Outcomes Among Study Participants With Pulmonary Fibrosis Stratified by Race/Ethnicity in the External Multicenter Validation (EMV) Cohort

eFigure 3. Distribution of Median Age at Outcomes Among Subjects With Pulmonary Fibrosis Aged 80 Years and Below in the External Multicenter Validation (EMV) Cohort Stratified by Age Group in Deciles and Race/Ethnicity

eFigure 4. Mean Age at Outcomes Among Study Participants Categorized by the Presence of Idiopathic Pulmonary Fibrosis (IPF) and Non-IPF Subtypes of Pulmonary Fibrosis and Substratified by Race/Ethnicity

eFigure 5. Mean Age at Outcomes Among Study Participants Categorized by the Presence of Connective Tissue Disease–Related Interstitial Lung Disease (CTD-ILD) and Non–CTD-ILD Subtypes of Pulmonary Fibrosis and Substratified by Race/Ethnicity

eFigure 6. Mean Age at Outcomes Among Study Participants Categorized by the Presence of Idiopathic Pulmonary Fibrosis (IPF) and Non-IPF Subtypes of Pulmonary Fibrosis and Substratified by Race/Ethnicity

eFigure 7. Mean Age at Outcomes Among Study Participants Categorized by the Presence of Connective Tissue Disease–Related Interstitial Lung Disease (CTD-ILD) and Non–CTD-ILD Subtypes of Pulmonary Fibrosis and Substratified by Race/Ethnicity

eFigure 8. Transplant-Free Survival Stratified by Race/Ethnicity for Participants With Pulmonary Fibrosis in the External Multicenter Validation Cohort

This supplemental material has been provided by the authors to give readers additional information about their work.

© 2023 Adegunsoye A et al. *JAMA Network Open*.

eTable 1. Baseline characteristics of the External Multicenter Validation (EMV) cohort stratified by site.

Characteristics*	UCHICAGO (n=1501)	UTSW (n=707)	UCSF (n=387)	UCD (n=293)
Age, years	63.6 (12.3)	63.5 (11.6)	69.1 (9.7)	70.2 (10.5)
Male	747 (49.8)	385 (54.5)	288 (74.4)	164 (56.0)
Ever Smoker	836 (57.2)	372 (52.6)	252 (65.1)	160 (54.6)
Body Mass Index	30.7 (6.5)	28.6 (5.9)	28.1 (5.1)	28.5 (6.4)
Lung Function				
FVC (% predicted)	66.3 (19.2)	67.1 (19.1)	69.4 (17.3)	71.6 (19.8)
DL_{CO} (% predicted)	55.1 (24.0)	46.1 (18.7)	47.9 (16.6)	51.0 (18.4)
GAP score	3.0 (1.7)	3.1 (1.5)	3.4 (1.4)	3.2 (1.4)
Race/Ethnicity				
Black	312 (21)	55 (8)	5 (1)	11 (4)
Hispanic	50 (3)	81 (11)	31 (8)	33 (11)
White	1139 (76)	571 (81)	351 (91)	249 (85)
PF sub-category				
IPF	418 (27.9)	306 (43.3)	327 (84.5)	92 (31.4)
CTD-ILD	269 (17.9)	154 (21.8)	1 (0.2)	67 (22.9)
fHP	173 (11.5)	92 (13.0)	58 (15.0)	49 (16.7)
Unclassifiable/Others	594 (39.6)	155 (21.9)	1 (0.2)	85 (29.0)
PF-specific Therapy				
Antifibrotic	162 (11.1)	167 (23.6)	128 (40.4)	--
Corticosteroids	810 (55.7)	361 (56.5)	82 (21.8)	--

*Categorical variables presented as n (%); continuous variables presented as means (SD). FVC=forced vital capacity; DL_{CO}=diffusing capacity of the lungs. ILD=interstitial lung disease; IPF=idiopathic pulmonary fibrosis; PF=pulmonary fibrosis; CTD-ILD=connective tissue disease associated-ILD; fHP=fibrotic hypersensitivity pneumonitis; unclassifiable/other ILD. GAP score = gender, age, physiology (FVC and DLCO) score. Data on PF-specific therapy unavailable for the UCD subgroup. UCHICAGO=University of Chicago; UTSW=University of Texas Southwestern; UCSF=University of California San Francisco; UCDAVIS=University of California Davis

© 2023 Adegunsoye A et al. *JAMA Network Open*.

eTable 2. Association of racial/ethnic categories with mortality in pulmonary fibrosis in the External Multicenter Validation (EMV) cohort.

Characteristics	BLACK	HISPANIC	WHITE	*P*-value
University of Chicago subpopulation	*(n=308)*	*(n=48)*	*(n=1111)*	
Unadjusted Hazard Ratio (95% CI)	0.62 (0.48-0.80)	--	*ref*	<0.001
	--	0.67 (0.37-1.23)	*ref*	0.20
Adjusted Hazard Ratio (95% CI) Model 1*	0.73 (0.56-0.94)	--	*ref*	0.02
	--	0.69 (0.38-1.26)	*ref*	0.23
Adjusted Hazard Ratio (95% CI) Model 2**	0.72 (0.56-0.94)	--	*ref*	0.02
	--	0.67 (0.37-1.22)	*ref*	0.19
University of Texas subpopulation	*(n=53)*	*(n=81)*	*(n=558)*	
Unadjusted Hazard Ratio (95% CI)	0.59 (0.39-0.89)	--	*ref*	0.01
	--	0.83 (0.60-1.14)	*ref*	0.25
Adjusted Hazard Ratio (95% CI) Model 1*	0.66 (0.43-1.02)	--	*ref*	0.06
	--	0.81 (0.59-1.12)	*ref*	0.20
Adjusted Hazard Ratio (95% CI) Model 2**	0.69 (0.45-1.06)	--	*ref*	0.09
	--	0.86 (0.62-1.19)	*ref*	0.35
University of California subpopulation	*(n=16)*	*(n=63)*	*(n=590)*	
Unadjusted Hazard Ratio (95% CI)	0.52 (0.22-1.27)	--	*ref*	0.15
	--	0.81 (0.55-1.20)	*ref*	0.30
Adjusted Hazard Ratio (95% CI) Model 1*	0.28 (0.11-1.69)	--	*ref*	0.006
	--	0.85 (0.57-1.26)	*ref*	0.41
Adjusted Hazard Ratio (95% CI) Model 2**	0.28 (0.11-0.70)	--	*ref*	0.006
	--	0.85 (0.57-1.26)	*ref*	0.42
Pooled population	*(n=383)*	*(n=195)*	*(n=2310)*	
Unadjusted Hazard Ratio (95% CI)	0.45 (0.37-0.55)	--	*ref*	<0.001
	--	1.01 (0.81-1.27)	*ref*	0.91
Adjusted Hazard Ratio (95% CI) Model 1*	0.68 (0.55-0.84)	--	*ref*	<0.001
	--	0.82 (0.65-1.03)	*ref*	0.09
Adjusted Hazard Ratio (95% CI) Model 2**	0.68 (0.55-0.84)	--	*ref*	<0.001
	--	0.83 (0.66-1.05)	*ref*	0.12

**Model 1 = Mortality hazard ratio computed using multivariable Cox regression models adjusting for age, sex, forced vital capacity, diffusing capacity of the lungs for carbon monoxide, interstitial lung disease subtype, and hospital center. **Model 2 = Mortality hazard ratio computed using multivariable Cox regression models adjusting*

for age, sex, forced vital capacity, diffusing capacity of the lungs for carbon monoxide, interstitial lung disease subtype, hospital center, and tobacco smoking.

© 2023 Adegunsoye A et al. *JAMA Network Open*.

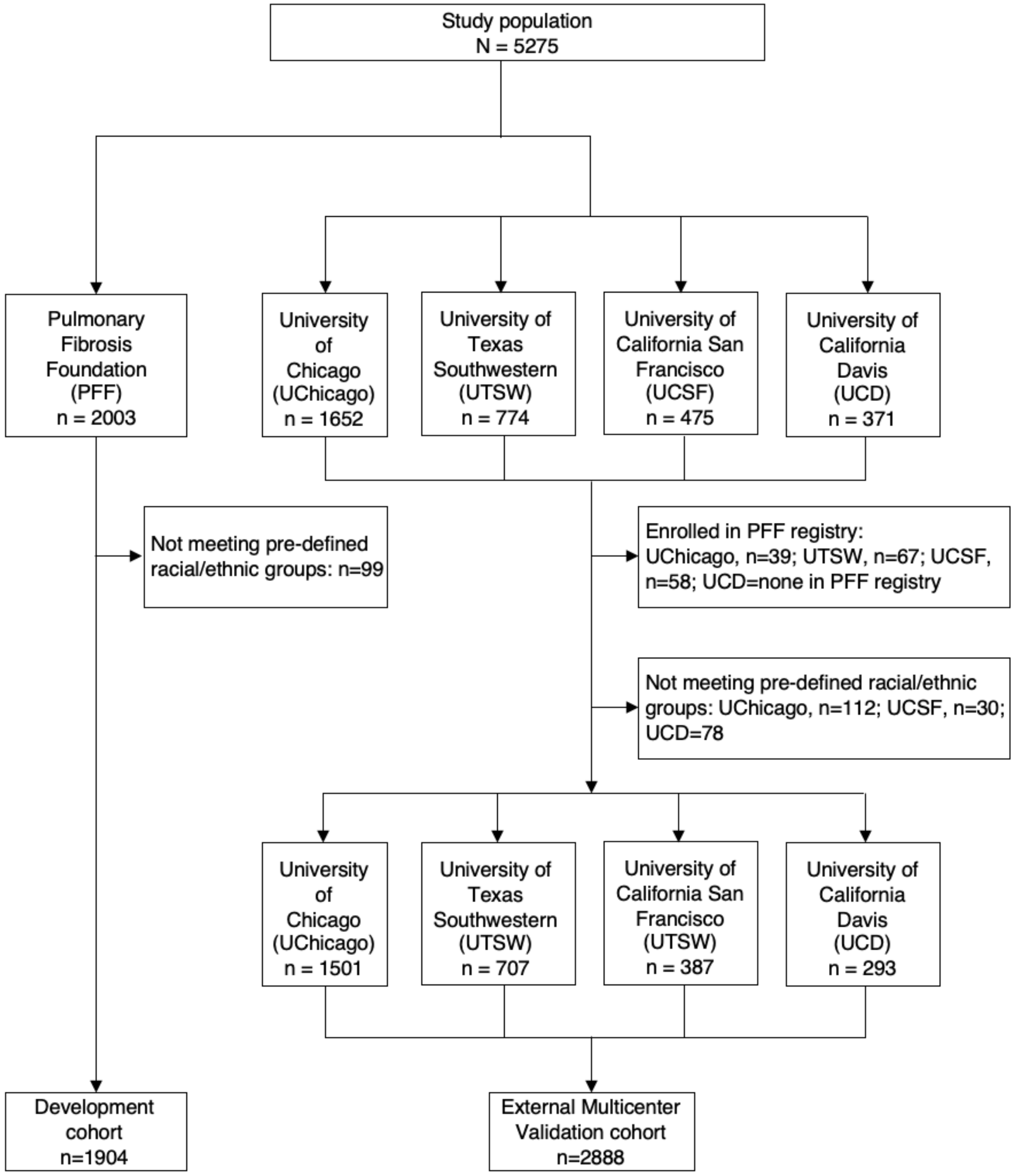

eFigure 1. STROBE diagram

© 2023 Adegunsoye A et al. *JAMA Network Open*.

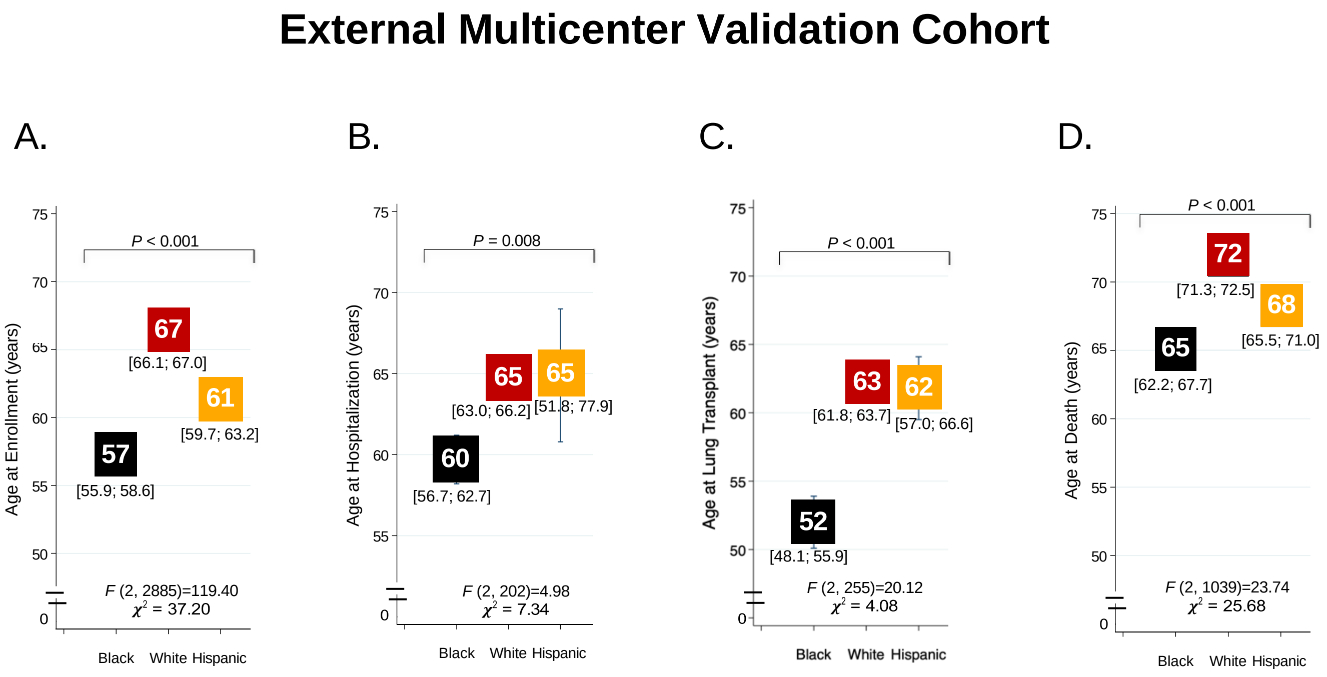

eFigure 2. Mean age at outcomes among study participants with pulmonary fibrosis stratified by race/ethnicity in the external multicenter validation (EMV) cohort. Significant differences in age at outcomes observed among study participants for **(A)** age at enrollment; **(B)** age at first hospitalization; **(C)** age at lung transplantation; and **(D)** age at death. *White, n=2310; Black, n=383; Hispanic, n=195. Thin vertical bars = standard error of the mean. 95% confidence interval depicted in brackets.* **P*-value for ANOVA statistical test comparing all three racial groups.

© 2023 Adegunsoye A et al. *JAMA Network Open*.

A.

Age at Diagnosis

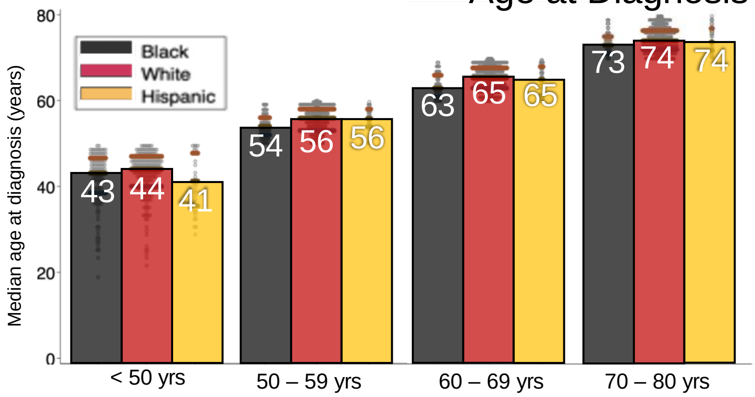

AGE GROUP	BLACK	WHITE	HISPANIC
< 50 yrs, median (IQR)	43 (8.1)	44 (7.0)	41 (12.3)
frequency, n	112	181	32
50 to 59 yrs, median (IQR)	54 (4.0)	56 (5.0)	56 (4.1)
frequency, n	101	361	47
60 to 69 yrs, median (IQR)	63 (5.0)	65 (4.7)	65 (6.7)
frequency, n	87	791	67
70 to 80 yrs, median (IQR)	73 (4.0)	74 (4.4)	74 (5.1)
frequency, n	62	763	38

B.

Age at First Hospitalization

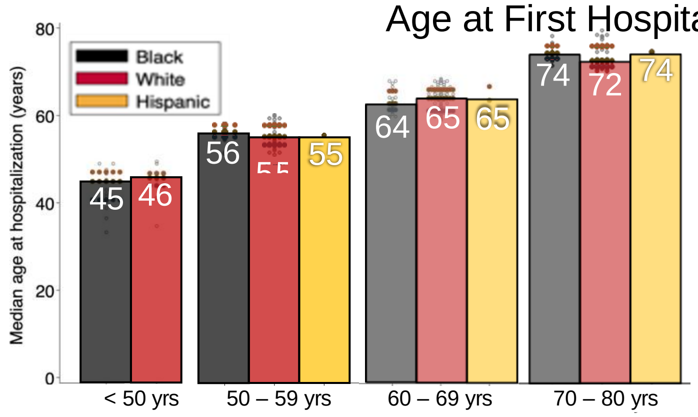

AGE GROUP	BLACK	WHITE	HISPANIC
< 50 yrs, median (IQR)	45 (6.4)	46 (2.8)	--
frequency, n	16	10	--
50 to 59 yrs, median (IQR)	56 (2.7)	55 (4.4)	55 (0.0)
frequency, n	20	24	1
60 to 69 yrs, median (IQR)	64 (4.4)	65 (5.0)	65 (6.5)
frequency, n	19	61	2
70 to 80 yrs, median (IQR)	74 (2.9)	72 (4.7)	74 (0.0)
frequency, n	9	33	1

C.

Age at Lung Transplantation

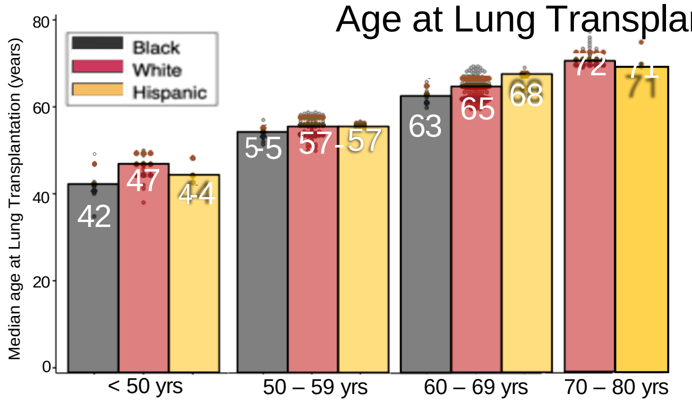

AGE GROUP	BLACK	WHITE	HISPANIC
< 50 yrs, median (IQR)	42 (6.2)	47 (5.0)	44 (5.6)
frequency, n	8	13	3
50 to 59 yrs, median (IQR)	55 (2.0)	57 (4.0)	57 (1.0)
frequency, n	9	53	4
60 to 69 yrs, median (IQR)	63 (3.9)	65 (4.8)	68 (3.5)
frequency, n	4	119	8
70 to 80 yrs, median (IQR)	--	72 (2.9)	71 (5.8)
frequency, n	--	33	3

D.

Age at Death

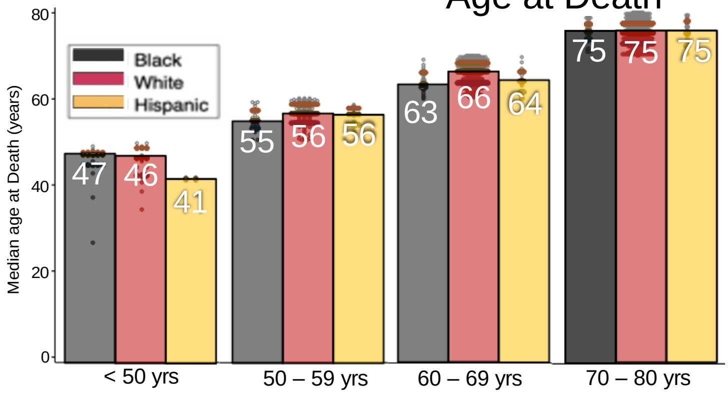

AGE GROUP	BLACK	WHITE	HISPANIC
< 50 yrs, median (IQR)	47 (3.0)	46 (6.4)	41 (0.4)
frequency, n	13	15	2
50 to 59 yrs, median (IQR)	55 (4.2)	56 (4.3)	56 (3.8)
frequency, n	16	71	13
60 to 69 yrs, median (IQR)	63 (3.4)	66 (4.6)	64 (4.7)
frequency, n	25	263	18
70 to 80 yrs, median (IQR)	75 (3.4)	75 (4.7)	75 (5.7)
frequency, n	24	378	21

eFigure 3. Distribution of median age at outcomes among subjects with pulmonary fibrosis aged 80 years and below in the external multicenter validation (EMV) cohort stratified by age group in deciles and race/ethnicity. Median ages at **(A)** diagnosis (n=2,888, *P*<0.0001); **(B)** first hospitalization (n=205/2,888, *P*<0.0001); **(C)** lung transplantation (n=257/2,888, *P*<0.0001); and **(D)**

© 2023 Adegunsoye A et al. *JAMA Network Open*.

death (n=1042/2,888, P<0.0001). *White = non-Hispanic White subjects; Black = non-Hispanic Black subjects; Hispanic = Hispanic subjects*.

© 2023 Adegunsoye A et al. *JAMA Network Open*.

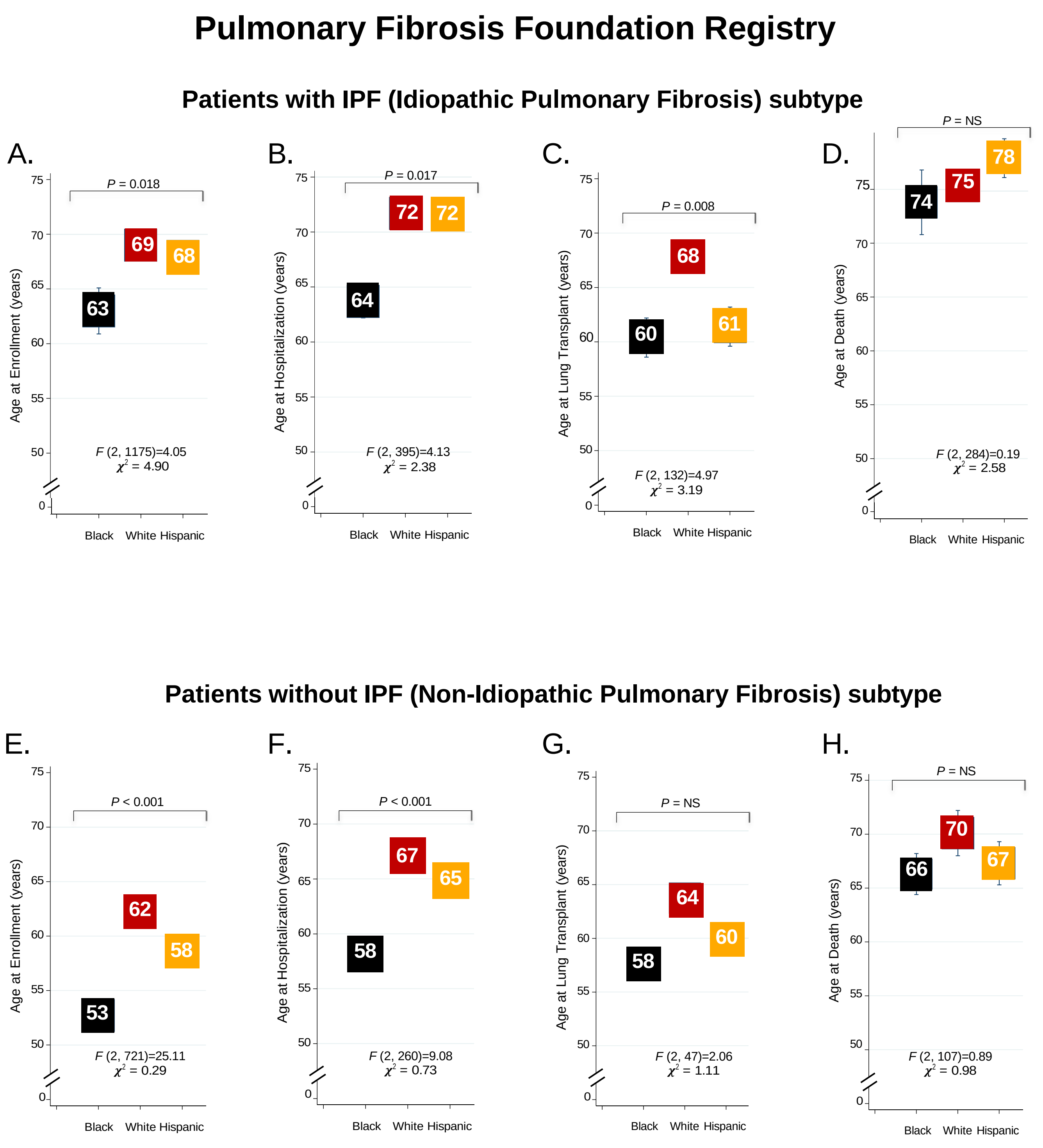

eFigure 4. Mean age at outcomes among study participants categorized by the presence of idiopathic pulmonary fibrosis (IPF) and non-IPF subtypes of pulmonary fibrosis and sub-stratified by race/ethnicity. Plot shows significant differences in age at outcomes among participants with IPF in the pulmonary fibrosis foundation registry (PFFR) for **(A)** age at enrollment; **(B)** age at first hospitalization; **(C)** age at lung transplantation; and **(D)** age at death; and among participants without IPF in the PFFR registry for **(E)** age at enrollment; **(F)** age at first hospitalization; **(G)** age at lung transplantation; and **(H)** age at death. PFFR cohort, *White = non-Hispanic White subjects, n=1675; Black = non-Hispanic Black subjects, n=105; Hispanic = Hispanic subjects, n=124.* EMV cohort, *White, n=2310; Black, n=383; Hispanic, n=195.* **P*-value for ANOVA statistical test comparing all three racial groups.

© 2023 Adegunsoye A et al. *JAMA Network Open*.

Pulmonary Fibrosis Foundation Registry

Patients with CTD-ILD (Connective Tissue Disease-related Interstitial Lung Disease) subtype

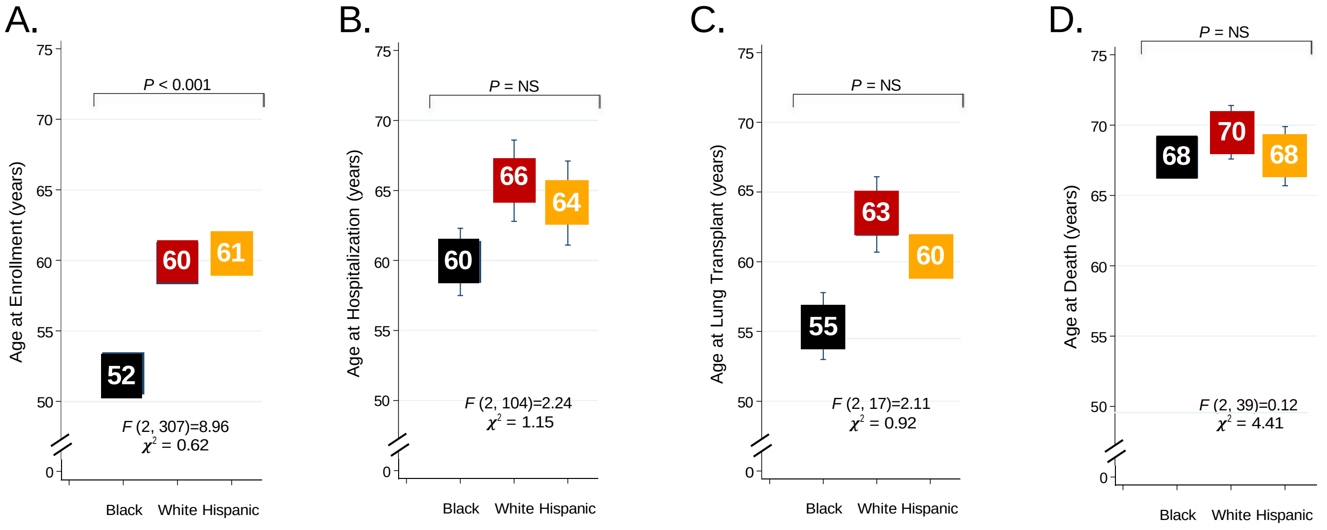

Patients without CTD-ILD (Non-Connective Tissue Disease-related Interstitial Lung Disease) subtype

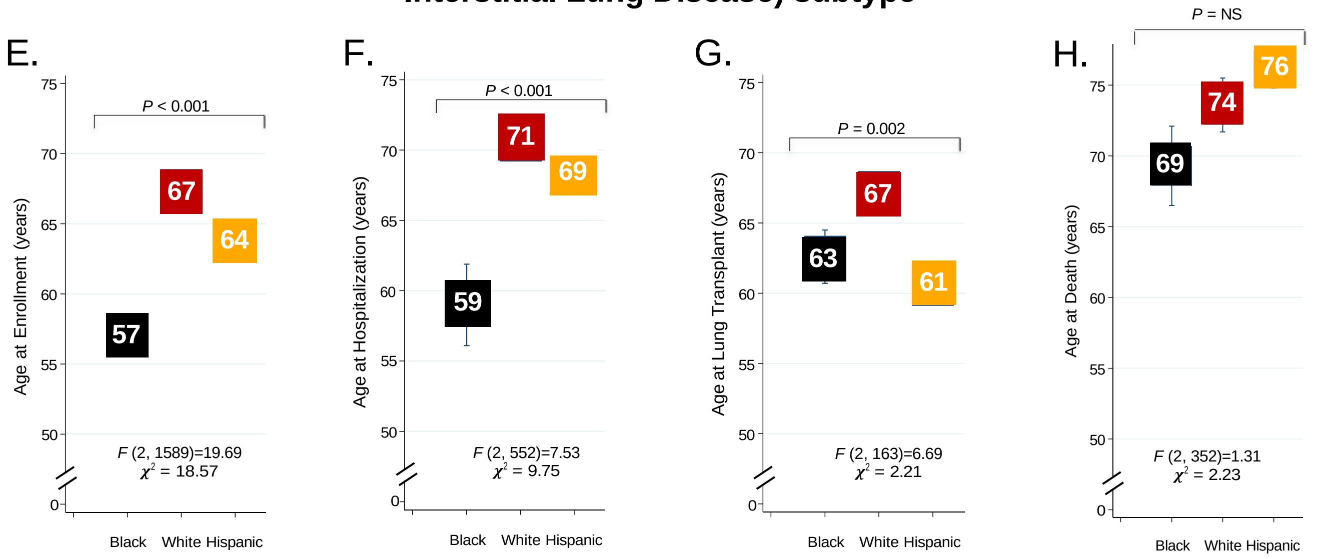

eFigure 5. Mean age at outcomes among study participants categorized by the presence of connective tissue disease-related interstitial lung disease (CTD-ILD) and non-CTD-ILD subtypes of pulmonary fibrosis and sub-stratified by race/ethnicity. Plot shows significant differences in age at outcomes among participants with CTD-ILD in the pulmonary fibrosis foundation registry (PFFR) for **(A)** age at enrollment; **(B)** age at first hospitalization; **(C)** age at lung transplantation; and **(D)** age at death; and among participants without CTD-ILD in the PFFR registry for **(E)** age at enrollment; **(F)** age at first hospitalization; **(G)** age at lung transplantation; and **(H)** age at death. PFFR cohort, *White = non-Hispanic White subjects, n=1675; Black = non-Hispanic Black subjects, n=105; Hispanic = Hispanic subjects, n=124.* EMV cohort, *White, n=2310; Black, n=383; Hispanic, n=195.* **P*-value for ANOVA statistical test comparing all three racial groups.

© 2023 Adegunsoye A et al. *JAMA Network Open*.

External Multicenter Validation Cohort

Patients with IPF (Idiopathic Pulmonary Fibrosis) subtype

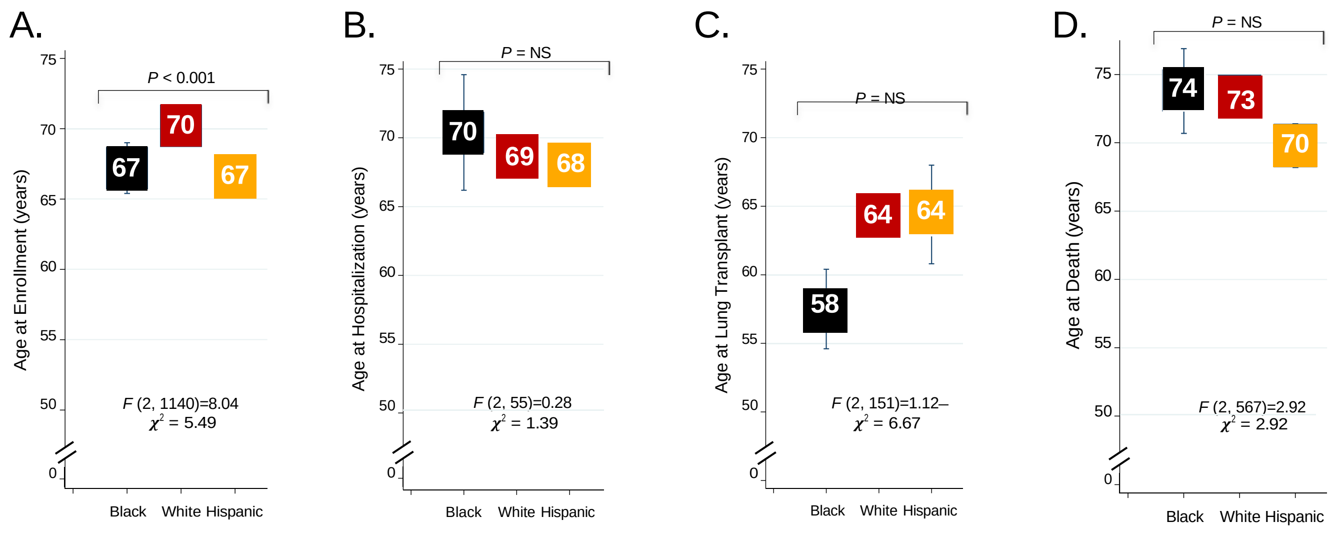

Patients without IPF (Idiopathic Pulmonary Fibrosis) subtype

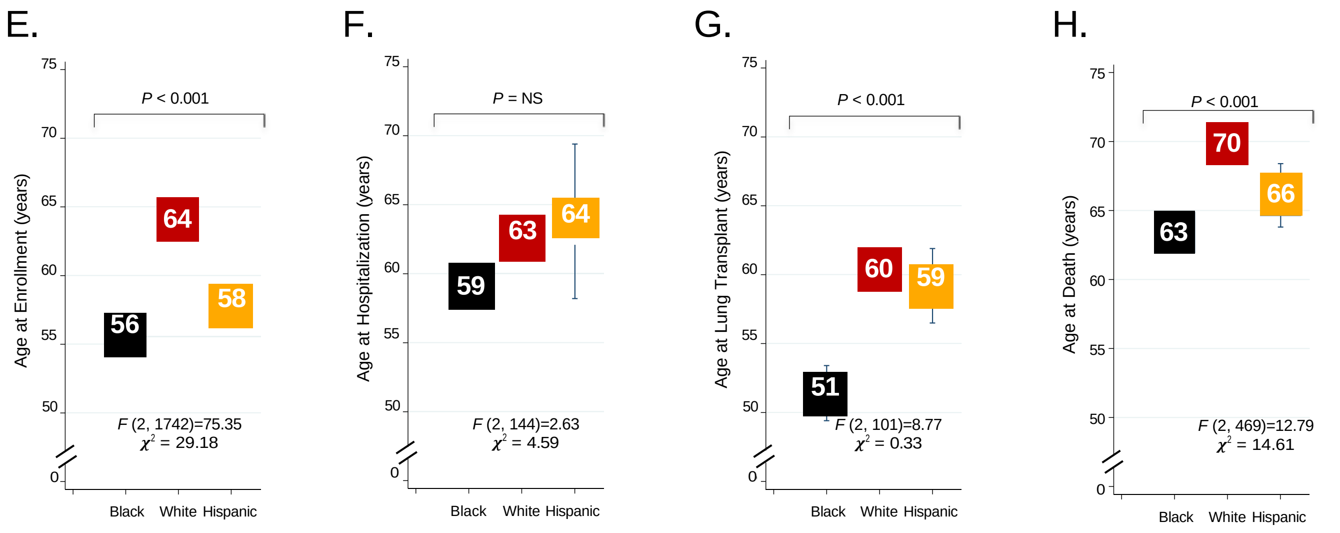

eFigure 6. Mean age at outcomes among study participants categorized by the presence of idiopathic pulmonary fibrosis (IPF) and non-IPF subtypes of pulmonary fibrosis and sub-stratified by race/ethnicity. Plot shows significant differences in age at outcomes among participants with IPF in the external multicenter validation (EMV) cohort for **(A)** age at enrollment; **(B)** age at first hospitalization; **(C)** age at lung transplantation; and **(D)** age at death; and among participants without IPF in the EMV registry for **(E)** age at enrollment; **(F)** age at first hospitalization; **(G)** age at lung transplantation; and **(H)** age at death. PFFR cohort, *White = non-Hispanic White subjects, n=1675; Black = non-Hispanic Black subjects, n=105; Hispanic = Hispanic subjects, n=124.* EMV cohort, *White, n=2310; Black, n=383; Hispanic, n=195. NS= not significant* *P-value for ANOVA statistical test comparing all three racial groups.

© 2023 Adegunsoye A et al. *JAMA Network Open*.

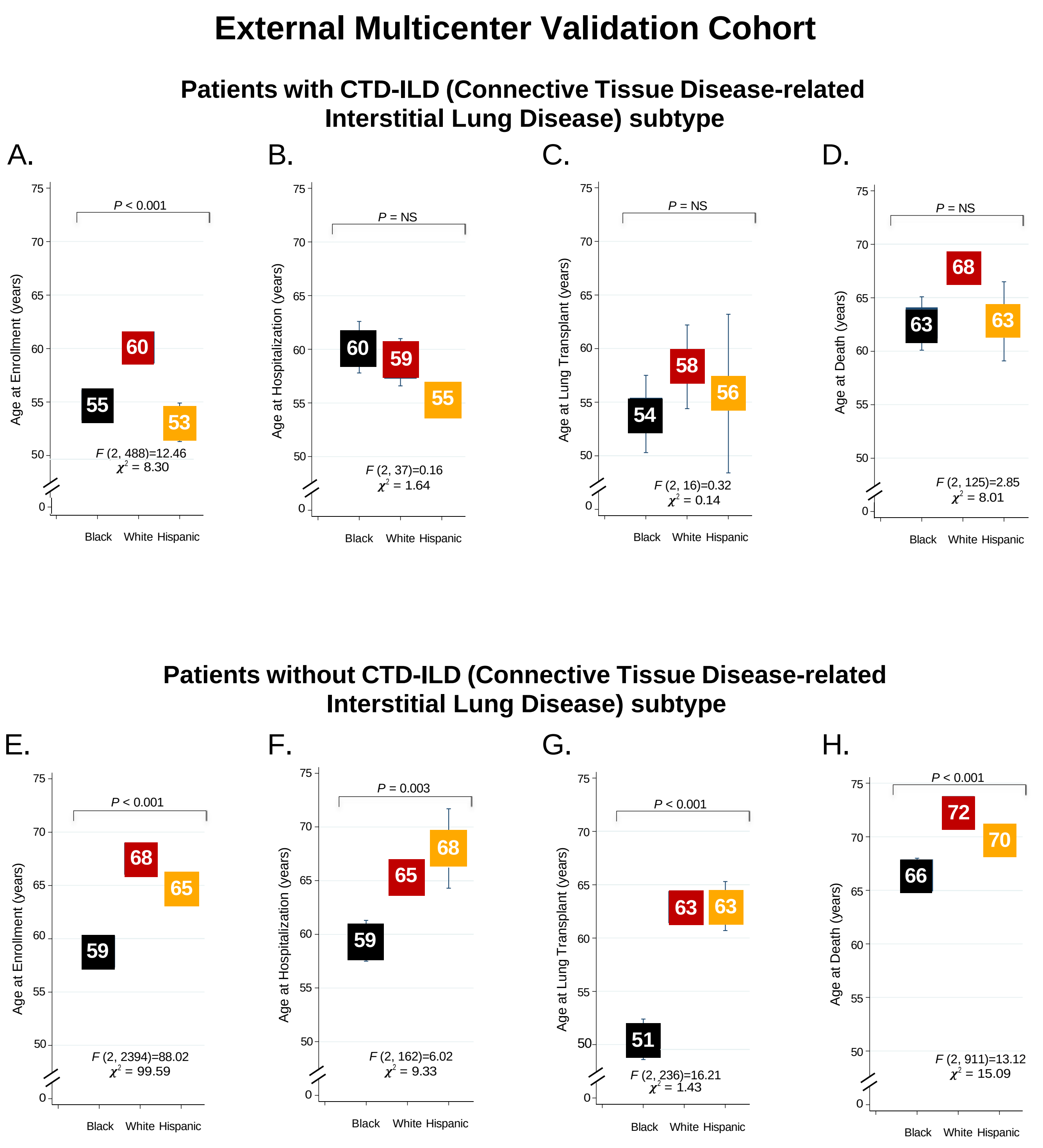

eFigure 7. Mean age at outcomes among study participants categorized by the presence of connective tissue disease-related interstitial lung disease (CTD-ILD) and non-CTD-ILD subtypes of pulmonary fibrosis and sub-stratified by race/ethnicity. Plot shows significant differences in age at outcomes among participants with CTD-ILD in the external multicenter validation (EMV) cohort for **(A)** age at enrollment; **(B)** age at first hospitalization; **(C)** age at lung transplantation; and **(D)** age at death; and among participants without CTD-ILD in the EMV registry for **(E)** age at enrollment; **(F)** age at first hospitalization; **(G)** age at lung transplantation; and **(H)** age at death. PFFR cohort, *White = non-Hispanic White subjects, n=1675; Black = non-Hispanic Black subjects, n=105; Hispanic = Hispanic subjects, n=124.* EMV cohort, *White, n=2310; Black, n=383; Hispanic, n=195.* **P*-value for ANOVA statistical test comparing all three racial groups.

© 2023 Adegunsoye A et al. *JAMA Network Open*.

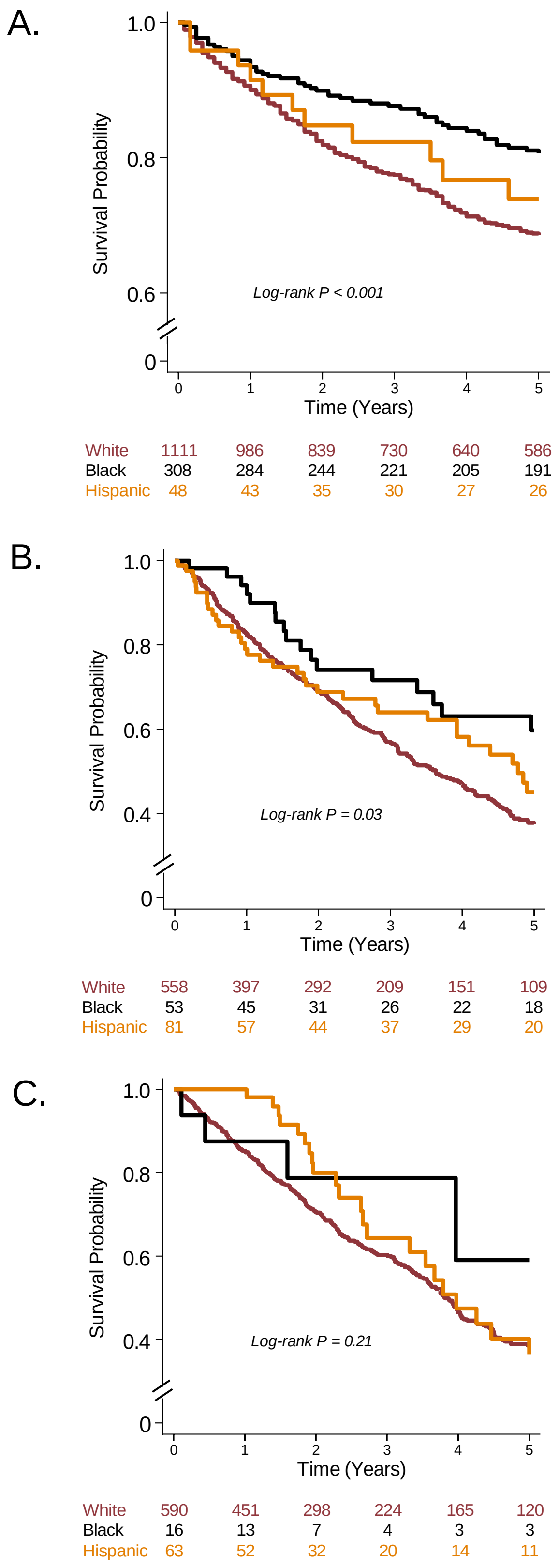

eFigure 8. Transplant-free survival stratified by race/ethnicity for participants with pulmonary fibrosis in the External Multicenter Validation Cohort (A) the University of Chicago subpopulation, (B) the University of Texas Southwestern subpopulation, and (C) the University of California subpopulation. *White = non-Hispanic White subjects; Black = non-Hispanic Black*

© 2023 Adegunsoye A et al. *JAMA Network Open*.

subjects; Hispanic = Hispanic subjects. P-value for log rank test comparing the survival distributions of all three racial groups.

© 2023 Adegunsoye A et al. *JAMA Network Open*.
