# Supplementary material for: Evaluation of Pulmonary Fibrosis Outcomes by Race and Ethnicity in US Adults
Source: JAMA Netw Open. Author manuscript; Available in PMC 2024 Apr 1. (PMC10984340; doi:10.1001/jamanetworkopen.2023.2427)
Supplement: Supplement 2 [file NIHMS1973253-supplement-Supplement_2.pdf]

Data Sharing Statement

Adegunsoye. Evaluation of Pulmonary Fibrosis Outcomes by Race and Ethnicity in US Adults. *JAMA Netw Open*. Published March 10, 2023. doi:10.1001/jamanetworkopen.2023.2427

Data
Data available: Yes
Data types: Deidentified participant data
How to access data: pffr-dcc-pm@umich.edu
When available: With publication

Supporting Documents
Document types: None

Additional Information
Who can access the data: Data will be available to researchers on reasonable request subject to restrictions from the Pulmonary Fibrosis Foundation. The pulmonary fibrosis external validation cohort data that support the findings of this study are not openly available due to reasons of clinical human data sensitivity and are available upon reasonable request from the corresponding author and the respective institutions.
Types of analyses: Research
Mechanisms of data availability: After approval of a proposal, with investigator support
